# School-based intervention on behavioral intention of adolescents regarding healthy diet in India

**DOI:** 10.3389/fpubh.2023.1094960

**Published:** 2023-02-09

**Authors:** Sweety Suman Jha, Madhumita Dobe, Chandrashekhar Taklikar, Arista Lahiri

**Affiliations:** ^1^Indian Institute of Technology Kharagpur, Kharagpur, West Bengal, India; ^2^Foundation for Actions and Innovations Towards Health Promotion, Kolkata, India; ^3^All India Institute of Hygiene and Public Health, Kolkata, West Bengal, India

**Keywords:** health behavior, healthy diet, health promotion, behavioral intention, Theory of Planned Behavior, adolescents

## Abstract

**Introduction:**

Adolescence is a distinctive period of life when intense physical, psychological, and cognitive development occurs. A healthy diet helps prevent various forms of malnutrition and non-communicable diseases (NCDs) like diabetes, heart disease, stroke, and cancer. The current study aimed to assess the change in behavioral intentions (measured based on the TPB) toward healthy dietary practices through health promotion intervention among adolescents studying in selected schools in an urban area of West Bengal, India

**Methods:**

The current study was a non-randomized controlled interventional study conducted among adolescents in either seventh, eighth, ninth, or tenth grades and aged between 12 and 16 years. Two-step cluster analysis with maximum likelihood estimation identified the intenders of a healthy diet. The intervention effect was measured using Relative Risk (RR) for being in the higher intention cluster through Generalized Linear Model (GLM) with a log-linear link under Poisson distribution assumptions and robust standard errors. A *P-*value of 0.05 or lesser was considered statistically significant.

**Results:**

There was no statistically significant difference in the mean score of “Attitude” in both groups. The mean score of “Subjective Norm” among the intervention group increased after intervention, and the difference was statistically significant. The mean score of “Perceived behavioral control” in the intervention group increased after the intervention, but the difference was statistically not significant. The intervention group's post-intervention proportion of intenders increased, and the difference was statistically significant. The relative risk of becoming an intender for healthy diet consumption in the Intervention group compared to the Control group was 2.07 (1.44–2.97).

**Conclusions:**

The intervention package effectively brought about a positive change in behavioral intention toward healthy dietary practices among adolescents. Model-based and construct-oriented intervention packages can be adopted in school-setting to promote behavioral intention toward a healthy diet.

## 1. Introduction

World Health Organization (WHO) and the United Nations (UN) defined adolescents as individuals in the 10–19 age group (1). Adolescence is when intense physical, psychological, and cognitive development occurs (2). In this phase of life, people become independent individuals, build up new relationships, develop social skills, and learn behaviors that will last forever in their life span (3). Adolescence is an age of opportunity for children and a pivotal time to build on their development in the first decade of life, to help them navigate risks and vulnerabilities, and to set them on the path to fulfilling their potential. Dietary Habits are the habitual decisions of individuals, groups, or cultures when choosing what food to eat. A healthy diet helps prevent various forms of malnutrition and non-communicable diseases (NCDs) like diabetes, heart disease, stroke, and cancer (4). The most significant health consequences of childhood overweight and obesity, which become primarily apparent in adulthood, include cardiovascular diseases (mainly heart disease and stroke), diabetes, musculoskeletal disorders, especially osteoarthritis, and certain types of cancer (5). The increasing level of overweight and obesity among children and adolescents is of grave concern, as it links childhood and adolescent obesity with the increased risk of obesity and morbidity in adulthood (6). Faulty dietary habits of skipping meals or eating junk food were associated with more number of participants under the obese or overweight category in a study conducted in India (7). The issue of healthy and unhealthy food consumption among adolescents is crucial, even considering the COVID-19 pandemic. The findings from one systematic review and meta-analysis conducted by Pourghazi et al. (8) stated that the impact of COVID-19 on children and adolescents eating habits was positive and negative, for example, a decrease in fast food, fruits, and vegetable consumption and an increase in snacks and sweet consumption. Both the changes may have a critical short- and long-term impact on adolescents' health.

In general, daily consumption of a diet with recommended proportions of staples like cereals, starchy tubers or roots, legumes, fruits and vegetables, and foods from animal sources is considered a healthy dietary habit (9). In an interventional study conducted by Menor-Rodriguez et al., the educational intervention reduced the levels of overweight and obesity in school children and improved their eating habits (10). Correa et al., in their study among Indian-origin adolescents, reported that all adolescents perceived foods high in vitamins and minerals as healthy (11). In a cross-sectional study, Kumar et al. found that 90% frequently consumed street foods, and 54% reported having overall poor eating habits (12). In a Quasi-experimental study among female students, a significant difference was noted between the two groups regarding the mean scores of attitudes, perceived behavioral control, and behavioral intention. In contrast, no significant difference regarding the mean score of subjective norms was found (13). Families, physicians, teachers, friends, society, and nutrition specialists are critical subjective norms in various studies (14–16).

Health promotion and education are one of several possible intervention strategies to address these various problems (17). It aims to increase awareness, expand knowledge, acquire skills and shape a health-oriented attitude of particular persons who are also perceived as components of society (18). To change behavior, one must first make adolescents aware of the consequences of their behavior. However, knowledge alone is not sufficient to change behavior. There are multiple models of individual health behavior, among which the Theory of Planned Behavior (TPB) identifies behavioral intention as the best predictor of behavior (19). The TPB can assess the behavioral intentions of adolescents toward a healthy diet by measuring the following constructs, i.e., “Attitude,” “Subjective norm,” and “Perceived behavioral control.” In a systematic review conducted by McDermott et al., it was concluded that TPB variables have medium to large associations with both intention and dietary patterns and further provide a guide for designing effective interventions (20).

### 1.1. The Theory of Planned Behavior

#### 1.1.1. Behavioral intention

The degree to which an individual formulates behavioral plans to attain a behavioral goal. In other words, it is the perceived likelihood of performing the behavior (21, 22).

#### 1.1.2. Attitude

Attitude implies the degree to which a person has a favorable or unfavorable evaluation of the behavior of interest. It entails a consideration of the outcomes of performing the behavior (21, 22).

#### 1.1.3. Subjective norm

The belief about whether most people approve or disapprove of the behavior is a subjective norm. It relates to a person's beliefs about whether peers and people of importance to the person think they should engage in the behavior (21, 22).

#### 1.1.4. Perceived behavioral control

A person's perception of the ease or difficulty of performing the behavior of interest is perceived behavioral control (21, 22). It is the perceived likelihood of occurrence of each facilitating or constraining condition and its perceived effect in making behavioral performance difficult or easy. It varies across situations and actions, which results in a person having varying perceptions of behavioral control depending on the situation.

Cost-effective interventions through clear behavioral intentions should be promoted early in life, especially during adolescence, the most formative stage of life (3). It can be conclusively stated that to prevent the development of risk factors (unhealthy behaviors) in adulthood, the ideal time of intervention is during adolescence, as it is easier to avoid the inculcation of unhealthy habits and facilitate change of practices among young children who are amenable to correction (23). An in-depth study of behavioral intentions and the positive effect of health promotion regarding healthy behavior, like healthy dietary practices among these young populations, may provide valuable insights for health administrators and school health authorities. This study aimed to assess the change in behavioral intentions (determined based on the TPB) toward healthy dietary practices following a school-based health promotion intervention among adolescents in an urban area of West Bengal, India.

## 2. Methods

### 2.1. Study design

A school-based non-randomized controlled interventional study with parallel group design was conducted in two co-educational English medium schools in a selected West Bengal municipal area. The data collection was done between March 2019 and January 2020.

### 2.2. Study participants

**Figure 1:**
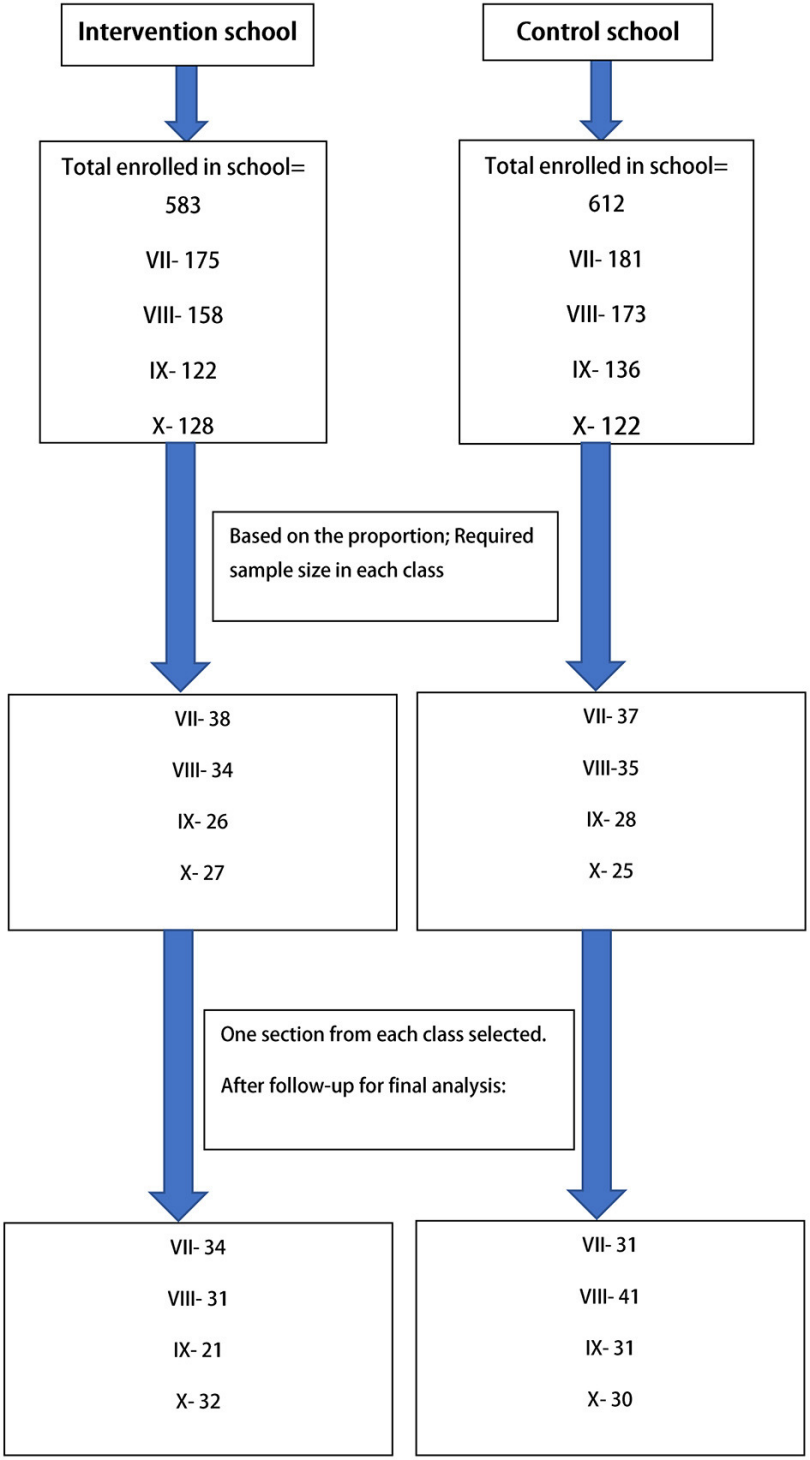
Flow diagram showing the selection of study participants.

Adolescents in seventh to tenth grades aged between 12 and 16 years, studying in the selected schools, whose parents gave consent and who provided assent for participation, were included in the study. Those who were absent at any phase of the study were excluded from the study. Multi-stage sampling was done. In the first stage, two schools were chosen based on enrolment and attendance (one for the intervention group and another for the control group) from the schools in the study area (Uttarpara-Kotrung Municipal area). In the selected schools, sections were chosen based on Probability Proportionate to Size (PPS) method in each grade. Then in the particular section, complete enumeration was done. The sample size was estimated using Fleiss' formula for difference in proportions for parallel group intervention study design (with both groups being equal in size) (24). For sample size calculation, a power of 80% and a confidence level of 95% were assumed. A design effect of 1.5 was used, and an attrition factor of 10% was considered. Based on a school-based study by Kebaili et al. (25), an increase in the proportion of intenders of healthy dietary habits (intention to eat vegetables regularly) among the control group taken as 5%, i.e., P_*control*_ = 0.05, and among intervention group 20%, i.e., P_*intervention*_ = 0.2. Therefore, using the Fleiss formula for difference in proportions, the minimum adequate sample size in each group was 76. With the design effect, the minimum sample size was 114 and using an attrition factor of 10%, the optimum sample size was 125 for each intervention and control group. Thus, the minimum sample size required in each group was 114; accounting for attrition, it was 125. Finally, after completing the post-intervention phase, 133 completed responses in the control group and 118 completed responses in the intervention group were obtained. Figure 1 depicts the selection of study participants.

Among the final 251 responses, a significant proportion of the intervention group (33.05%) belonged to the age group of 15 years, and the control group (37.60%) belonged to the age group of 14 years. Most participants in the intervention group (67.80%) were boys, whereas most participants (50.38%) were girls in the control group. The socio-demographic characteristics of the participants are given in Table 1.

**Table 1:** Socio-demographic characteristics of the participants.

**Socio-demographic characteristics**		**Intervention** **(*****n*** = **118)**		**Control** **(n** = **133)**		**Total** **(n** = **251)**		***P*-value**
		***N***	**%**	***N***	**%**	***N***	**%**	
Age (in completed years)	12	9	7.63	10	7.52	19	7.57	0.329
	13	21	17.80	32	24.06	53	21.11	
	14	37	31.35	50	37.60	87	34.66	
	15	39	33.05	30	22.55	69	27.49	
	16	12	10.17	11	8.27	23	9.17	
Gender	Boys	80	67.80	66	49.62	146	58.17	0.004
	Girls	38	32.20	67	50.38	105	41.83	
Religion	Hinduism	111	94.06	126	94.74	237	94.42	0.974
	Islam	4	3.39	4	3.00	8	3.19	
	Others^*^	3	2.55	3	2.26	6	2.39	
Type of family	Nuclear	70	59.32	94	70.68	164	65.33	0.059
	Joint	48	40.68	39	29.32	87	34.67	

“n” indicates the total participants in respective groups, “N” denotes the numbers in each category, and “%” denotes the respective column percentages. P-values are obtained by Chi-squared tests. ^*^Others include Jainism and Sikhism.

### 2.3. Measurements

Behavioral intention toward consumption of a healthy diet was determined utilizing the constructs from the TPB: (i) “Attitude” consisting of domains such as Behavioral belief and Evaluation of behavioral outcome, (ii) “Subjective norm” consisting of Normative beliefs and Motivation to comply, and (iii) “Perceived behavioral control” (PBC) consisting of Control beliefs and Perceived power. Items for intention measurement and identifying intenders were selected through elicitation interviews. These interviews were conducted based on the elicitation interview guide, which focused on the TPB's constructs. Elicitation interviews were done among 20 students of classes VII to X in a separate English medium co-educational school of the study area. The attitude was measured through the items: Sufficiency in consumption of vitamins and minerals, consumption of oily food, Sufficient protein intake, Obesity/overweight, Healthy life span, Taste of Food, and Cost of Food. For Subjective Norms, the items were: Mother, Father, Relatives/other family members, Friends/peers, Teachers, Contents of television, and Contents/discussions in social media. For the PBC construct, the items were as follows: choosing a healthy diet even when hungry, Not choosing unhealthy food even if they are tasteful, choosing to eat a healthy diet even when depressed or sad, opting to eat a healthy diet even when junk food is readily available, choosing to eat a healthy diet even during celebrations or parties, choosing to eat a healthy diet even while visiting any mall/theater, choosing to eat a healthy diet while traveling. Each item was measured on all three constructs through their respective domains using a dichotomous (Agree-Disagree) response. In this study, the intention was not directly computed; instead, the participants with higher intentions (the intenders) were identified in pre- and post-intervention phases through a combination of construct-wise measurements.

### 2.4. Instruments

A pre-designed, pretested and validated questionnaire was used to assess behavioral intention. After initial preparation, the questionnaire was reviewed by a group of 5 experts who made necessary corrections. Pretesting of the questionnaire was done among 40 students. Cronbach's alpha measured internal consistency reliability construct-wise with values ranging between 0.79 and 0.86. The questionnaire consisted of socio-demographic characteristics and the three constructs of TPB that determined the intention to consume a healthy diet (refer to Supplementary material).

The intervention booklet and demonstration material were designed, developed, and pretested based on the construct-wise findings from elicitation interviews on healthy dietary practices. The conclusions of the pre-intervention survey were utilized in refining the content of the intervention tools. The intervention booklet contained information on different compositions of a healthy diet, accessible and affordable healthy food items, and the importance of developing and maintaining healthy dietary habits. During the interactive sessions, discussions were conducted on common myths and beliefs regarding healthy and unhealthy foods. Thrust was given to facilitating the enablers and overcoming the barriers to healthy dietary practices. Normative influencers and their role in motivating the participants to adopt healthy dietary habits were also discussed. The intervention tools were prepared with subject experts, following standard guidelines on healthy nutritional practices. The intervention materials were also pretested before implementation.

### 2.5. Procedures

Baseline data were collected from the students of grades VII–X of both the intervention and the control groups with the help of the self-administered questionnaire after obtaining assent. Each participant was allotted an Identification (ID) code, which was further used to associate the data from the same participant upon follow-up. After baseline data collection, the intervention was provided to the intervention group. The intervention was imparted grade-wise, i.e., students from a particular grade were intervened at a time. Booklets were distributed among the participants, and then lectures were conducted based on the booklet's contents. Pre-designed computer-based slides were used as visual aids for pointing out the existing disconnects between their thoughts and action. In between lectures and demonstrations, interactive sessions and discussions were also conducted. After completion of the sessions, queries or doubts were clarified through a question-and-answer session. Follow-up data were collected 3 months after the conclusion of the intervention. Follow-up data were also collected from the control group using the same questionnaire but without intervention. At the end of follow-up data collection, health education interventions were conducted among the control group during the study period in the same manner as in the intervention group.

### 2.6. Analysis

Pre-intervention and post-intervention data entered in a spreadsheet were linked through the unique codes. The cleaned dataset was used for statistical analysis using Statistical Package for Social Sciences (SPSS) software version 21 (IBM, Chicago, IL, USA). For computation purposes, items were coded 1 and 2, with 2 indicating a favorable response toward healthier habits. Negatively framed items were reverse-coded while maintaining the directionality of scoring, i.e., a favorable response was coded higher. Construct scores were calculated by multiplying the scores for the contributing domains (21), e.g., “Attitude” was calculated by multiplying the Behavioral belief score and evaluation of behavioral outcome score. A similar calculation was done for “Subjective Norm” and “Perceived Behavioral Control.” A linear combination of the responses of the respective item sets computed individual domain-specific scores. Intenders (i.e., those having the latent variable “Behavioral intention”) were identified by applying two-step cluster analysis with maximum likelihood estimation based on the calculated Attitude, Subjective norm, and Perceived behavioral control scores, separately for pre-intervention and post-intervention phases. Both intervention and control group measurements were taken for the cluster analysis. Those with a higher mean score in the constructs were considered intenders, and the remaining were non-intenders (refer to Table 2). Using the Silhouette measure, cluster analyses were found to be statistically adequate. The item-specific responses and proportions of intenders were compared between the pre-intervention and post-intervention phases in both the study groups.

**Table 2:** Description of clusters in Pre-intervention and post-intervention phases as per the defining constructs.

**Model constructs**	**Pre-intervention** ^**a**^		**Post-intervention** ^**b**^	
	**Cluster 1: Higher intention [*n =* 148]**	**Cluster 2: Lower intention [*n =* 103]**	**Cluster 1: Higher intention [*n =* 134]**	**Cluster 2: Lower intention [*n =* 116]**
Attitude	139.31 (±27.15)	130.64 (±30.83)	154.98 (±18.03)	119.18 (±25.41)
Subjective Norm	166.44 (±19.85)	109.35 (±18.92)	178.18 (±16.80)	128.28 (±25.75)
Perceived Behavioral Control	108.07 (±27.60)	90.23 (±24.41)	112.1 (±31.54)	109.33 (±28.21)

“n” indicates the number of participants in each of the identified clusters, values within parentheses in each of the cells represent the respective standard deviations. ^a^No outliers were detected; the average silhouette measure of cluster cohesion and separation was 0.5. ^b^One outlier was detected and subsequently excluded from the analysis; the average silhouette measure of cluster cohesion and separation was 0.4.

The Chi-square test was employed to test for any statistically significant difference in each item in the two study groups before and after the intervention phase. The construct scores were compared (pre- vs. post-intervention) with the help of paired *t*-tests in each study group. The intervention effect was measured using Relative Risk (RR) for being in the higher intention cluster through Generalized Linear Model (GLM) with the log-linear link under Poisson distribution assumptions and robust standard errors. The model was adjusted for the effects of gender, the baseline (pre-intervention) intention cluster, and their interaction with the receipt of intervention (i.e., study groups). The model was statistically significant (P_χ2_ <0.001). A two-tailed *P-*value of 0.05 or less was considered statistically significant in all the statistical techniques.

### 2.7. Ethical consideration

Approval for the study was obtained from the Institutional Ethics Committee of All India Institute of Hygiene and Public Health, Kolkata. Permission from the head of the schools was taken before data collection. Informed written assent was obtained from the participants, and informed written consent was obtained from the guardian.

## 3. Results

### 3.1. Measurement of intention toward healthy dietary practices

Regarding the choice of food based on its cost, among intervention group participants, there was a significant difference post-intervention in their behavior belief and evaluation of behavioral outcome (Table 3). Relatives/other family members were the important normative influencers among the intervention group participants after the intervention (Table 4). The intervention group participants had greater control belief and perceived power for “opting to eat a healthy diet even when junk food is easily available” after the intervention (Table 5).

**Table 3:** Pre- and post-intervention comparison of attitudes regarding consumption of healthy diet among intervention and control groups.

**Issues**	**Domain**	**Intervention (*****n*** = **118)**			**Control (*****n*** = **133)**		
		**Pre-intervention**	**Post-intervention**	**P-Value** ^a^	**Pre-intervention**	**Post-intervention**	**P-value** ^a^
Sufficiency in consumption of vitamins and minerals	Behavioral belief	88 (74.58)	89 (75.42)	0.881	113 (84.96)	98 (73.68)	0.023
	Evaluation of behavioral outcome	36 (30.51)	54 (45.76)	0.016	102 (76.69)	91 (68.42)	0.131
Consumption of oily food	Behavioral belief	98 (83.05)	99 (83.90)	0.861	129 (96.99)	122 (91.73)	0.063
	Evaluation of behavioral outcome	86 (72.88)	99 (83.90)	0.040	119 (89.47)	117 (87.97)	0.698
Sufficient protein intake	Behavioral belief	85 (72.03)	97 (82.20)	0.063	110 (82.71)	125 (93.98)	0.004
	Evaluation of behavioral outcome	52 (44.07)	49 (41.53)	0.693	102 (76.69)	99 (74.44)	0.669
Obesity/overweight	Behavioral belief	87 (73.73)	86 (72.88)	0.883	112 (84.21)	118 (88.72)	0.282
	Evaluation of behavioral outcome	87 (73.73)	95 (80.51)	0.215	119 (89.47)	118 (88.72)	0.844
Healthy life span	Behavioral belief	91 (77.12)	92 (77.97)	0.876	114 (85.71)	114 (85.71)	1.000
	Evaluation of behavioral outcome	85 (72.03)	96 (81.36)	0.090	102 (76.69)	108 (81.20)	0.367
Taste of Food	Behavioral belief	56 (47.46)	35 (29.66)	0.005	81 (60.90)	73 (54.89)	0.320
	Evaluation of behavioral outcome	42 (35.59)	44 (37.29)	0.787	71 (53.38)	54 (40.60)	0.037
Cost of Food	Behavioral belief	61 (51.69)	44 (37.29)	0.026	66 (49.62)	82 (61.65)	0.048
	Evaluation of behavioral outcome	72 (61.02)	38 (32.20)	0.000	101 (75.94)	99 (74.44)	0.776
Attitude Mean score [SD]		123.68 [±24.54]	123.11 [±27.73]	0.841 ^b^	146.47 [±28.46]	146.77 [±24.52]	0.925 ^b^

“n” represents the number of completed responses in each group. SD, Standard deviation of Perceived Behavioral Control score. Values within parentheses represent respective column-percentages. Values within square-brackets represent the SD. ^a^P-values calculated by chi-squared test except in ^b^. ^b^P-values were calculated by paired t-test.

**Table 4:** Pre- and post-intervention comparison of subjective norms regarding consumption of healthy diet among intervention and control groups.

**Influencers**	**Domain**	**Intervention (*****n =*** **118)**			**Control (*****n =*** **133)**		
		**Pre-intervention**	**Post-intervention**	**P-value** ^a^	**Pre-intervention**	**Post-intervention**	**P-value** ^a^
Mother	Normative beliefs	104 (88.14)	111 (94.07)	0.110	126 (94.74)	124 (93.23)	0.606
	Motivation to comply	91 (77.12)	97 (82.20)	0.332	117 (87.97)	123 (92.48)	0.215
Father	Normative beliefs	99 (83.90)	110 (93.22)	0.024	116 (87.22)	116 (87.22)	1.000
	Motivation to comply	92 (77.97)	90 (76.27)	0.757	100 (75.19)	104 (78.20)	0.562
Relatives/other family members	Normative beliefs	79 (66.95)	100 (84.75)	0.001	110 (82.71)	103 (77.44)	0.283
	Motivation to comply	73 (61.86)	92 (77.97)	0.007	99 (74.44)	106 (79.70)	0.307
Friends/peers	Normative beliefs	70 (59.32)	54 (45.76)	0.037	92 (69.17)	87 (65.41)	0.513
	Motivation to comply	64 (54.24)	64 (54.24)	1.000	80 (60.15)	80 (60.15)	1.000
Teachers	Normative beliefs	102 (86.44)	96 (81.36)	0.288	117 (87.97)	118 (88.72)	0.848
	Motivation to comply	81 (68.64)	84 (71.19)	0.670	96 (72.18)	98 (73.68)	0.783
Contents of television	Normative beliefs	75 (63.56)	66 (55.93)	0.232	74 (55.64)	77 (57.89)	0.710
	Motivation to comply	77 (65.25)	75 (63.56)	0.786	73 (54.89)	98 (73.68)	0.001
Contents/discussions in the social media	Normative beliefs	65 (55.08)	76 (64.41)	0.144	67 (50.38)	96 (72.18)	0.000
	Motivation to comply	50 (42.37)	82 (69.49)	0.000	80 (60.15)	103 (77.44)	0.002
Subjective Norm Mean score [SD]		138.88 [±32.72]	146.83 [±29.70]	0.028^b^	146.68 [±35.18]	155.04 [±36.10]	0.060^b^

“n” represents the number of completed responses in each group. SD, Standard deviation of Perceived Behavioral Control score. Values within parentheses represent respective column-percentages. Values within square-brackets represent the SD. ^a^P-values calculated by chi-squared test except in ^b^. ^b^P-values were calculated by paired t-test.

**Table 5:** Pre- and post-intervention comparison of perceived behavioral control regarding consumption of healthy diet among intervention and control groups.

**Situations**	**Domain**	**Intervention (*****n =*** **118)**			**Control (*****n =*** **133)**		
		**Pre-intervention**	**Post-intervention**	**P-value** ^a^	**Pre-intervention**	**Post-intervention**	**P-value** ^a^
Choosing a healthy diet even when hungry	Control belief	83 (70.34)	96 (81.36)	0.048	96 (72.18)	98 (73.68)	0.783
	Perceived power	79 (66.95)	63 (53.39)	0.033	66 (49.62)	78 (58.65)	0.140
Not choosing unhealthy food even if they are tasteful	Control belief	46 (38.98)	66 (55.93)	0.009	45 (33.83)	48 (36.09)	0.700
	Perceived power	50 (42.37)	60 (50.85)	0.192	65 (48.87)	68 (51.13)	0.713
Choosing to eat a healthy diet even when depressed or sad	Control belief	57 (48.31)	50 (42.37)	0.360	67 (50.38)	56 (42.11)	0.176
	Perceived power	61 (51.69)	64 (54.24)	0.696	75 (56.39)	59 (44.36)	0.050
Opting to eat a healthy diet even when junk food is easily available	Control belief	47 (39.83)	69 (58.47)	0.004	57 (42.86)	72 (54.14)	0.066
	Perceived power	47 (39.83)	80 (67.80)	0.000	51 (38.35)	84 (63.16)	0.000
Choosing to eat a healthy diet even during celebrations or parties	Control belief	48 (40.68)	56 (47.46)	0.294	37 (27.82)	64 (48.12)	0.001
	Perceived power	43 (36.44)	52 (44.07)	0.232	44 (33.08)	71 (53.38)	0.001
Choosing to eat a healthy diet even while visiting any mall/multiplex	Control belief	52 (44.07)	62 (52.54)	0.193	33 (24.81)	64 (48.12)	0.000
	Perceived power	60 (50.85)	40 (33.90)	0.008	48 (36.09)	70 (52.63)	0.007
Choosing to eat a healthy diet while traveling	Control belief	51 (43.22)	37 (31.36)	0.059	58 (43.61)	80 (60.15)	0.007
	Perceived power	46 (38.98)	57 (48.31)	0.149	69 (51.88)	75 (56.39)	0.460
Perceived behavioral control Mean Score [SD]		103.37 [±23.10]	108.11 [±26.08]	0.137^b^	98.43 [±31.15]	114.34 [±36.75]	0.000^b^

“n” represents the number of completed responses in each group. SD, Standard deviation of Perceived Behavioral Control score. Values within parentheses represent respective column-percentages. Values within square-brackets represent the SD. ^a^P-values calculated by chi-squared test except in ^b^. ^b^P-values were calculated by paired t-test.

### 3.2. Intenders and non-intenders of healthy dietary practices

**Figure 2:**
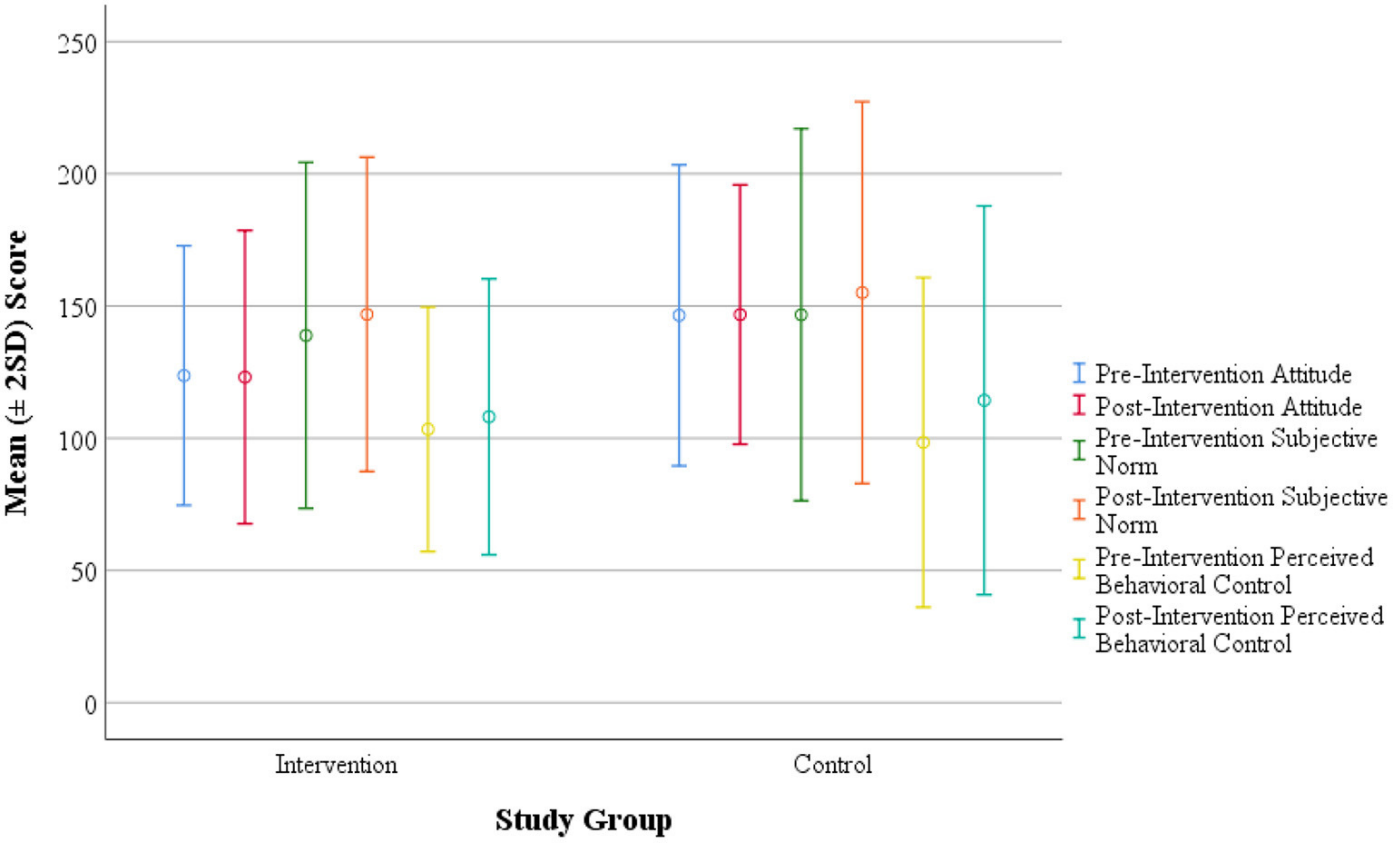
Score distribution of intervention and control groups with respect to Attitude, Subjective Norm and Perceived Behavioral Control (constructs) toward healthy dietary practices. The circles indicate the mean value computed, and the bars represent two standard deviation (2SD) around the mean.

In both the intervention and control groups, the Subjective norm and Perceived behavioral control mean scores increased in the post-intervention phase with a broader range of scores (Figure 2). The RR of becoming an intender for healthy diet consumption in the Intervention group compared to the Control group was 2.07 (1.44–2.97). Table 6 represents the generalized linear model showing the effect of intervention in improving intention.

**Table 6:** General linear model showing different factors of improved intention for healthy dietary behavior.

**Factors for becoming an intender for healthy dietary behavior**	**aRR (95% CI)**	***P*-value**
**Intervention (Ref.: Control group)**		
Intervention group	2.07 (1.44–2.97)	0.000
**Pre-Intervention Intention (Ref.: High intention cluster)**		
Low Intention Cluster	1.30 (0.97–1.74)	0.076
**Gender (Ref.: Girls)**		
Boys	1.18 (0.88–1.60)	0.270

aRR, Adjusted Relative Risk; CI, Confidence interval; P-values obtained through Generalized Linear Model (GLM) with the log-linear link under Poisson distribution function and robust standard errors: Akaike's Information Criterion (AIC) and Bayesian Information Criterion (BIC) were 394.646 and 415.774, respectively, with the scale parameter for the model set at 1. The model has been adjusted for the statistically significant two-way interaction sets: gender-study groups and pre-intervention cluster-study groups, with model intercept: 0.47 (95% CI: 0.35–0.62). The model was found to be an appropriate improvement over the intercept-only model (P-value < 0.001).

## 4. Discussion

In the study, the mean score of “Subjective Norm” among the intervention group increased after intervention, and the difference was statistically significant. The intervention group's post-intervention proportion of intenders increased, and the difference was statistically significant. The RR of becoming an intender following intervention was 2.07 (1.44–2.97) compared to the control group.

In the current study, there was an increase in the proportion of respondents in the intervention group who considered “Insufficient intake of vitamins and minerals on health” as bad. This finding was in accordance with a qualitative study conducted by Correa et al. (11) among Indian-origin adolescents. It was reported that all adolescents perceived foods high in vitamins and minerals as healthy. In the present study for the item “Oily food has a harmful effect on health,” there was an increased agreement among the intervention group, but it was statistically not significant. Statistically, a significant difference was observed in the increase in the proportion of respondents who considered the “Effects of oily food on health is bad” after the intervention. This finding aligned with the cluster randomized controlled trial conducted by Ochoa-Aviles et al. (26) among 1,430 Ecuadorian adolescents. The intervention group consumed lower quantities of unhealthy snacks like oily food. A cross-sectional study done in India by Kumar et al. (12) reported that 90% of adolescents frequently consumed street foods. In the intervention group, the mean score of “Attitude” decreased slightly following the intervention, although the difference was statistically not significant. In an interventional study from India conducted by Anand et al. (27), it was observed that the participants in the intervention group showed significant improvement in “Attitude” after the intervention. Dhauvadel et al. (28), in their interventional study conducted in Nepal, stated that the Education package was reported to be effective in changing the “Attitude” toward healthy eating behavior among adolescents.

Relatives/other family members were critical normative influencers among the intervention group participants. In a study conducted among Danish adolescents by Gronhoj et al. (16), it was observed that the highest “subjective norms” were family members, teachers, and television programs. For the item “Even when junk food is available, I find it easy to opt for a healthy diet,” there was an increase in the proportion of participants who agreed in both the intervention and control groups. Still, the difference was statistically significant only for the intervention group. In the intervention group, there was an increase in the proportion of agreement for the item “Can choose to eat a healthy diet even when junk food is easily available” after the intervention, and the difference was statistically significant. These findings in the present study revealed that intervention was adequate for the participants to develop and have more control over healthy eating behavior. This finding was consistent with the conclusions of the study done by Correa et al. (11), where it was mentioned that facilitators to healthy eating were personal preferences for healthy foods. However, a recent study (2021) noted that despite personal preferences and motivations, COVID-19-related lockdown had a menacing effect on practicing healthy dietary behavior (29).

As shown in the results, the intervention significantly improved the intention toward eating a healthy diet. This observation was in consonance with the finding from the study done by Dhauvadel et al. (28), where the Education package was reported to be effective in changing the intention toward healthy eating behavior. In contrast to the current study's finding, Poelman et al. (30), in the inquiry among Australian school children, stated that their intervention did not affect behavioral intentions. However, a systematic review by Bel-Serrat et al. (31) identified the importance of targeted behavioral interventions in improving adolescent dietary behaviors. Thus, designing appropriate school-based health promotion and education interventions relying on the assessment of behavioral intention among school students can lead to healthier children fostering regular healthy dietary practices in their homes and communities. Similarly, Char et al. (32), in their systematic review focused on South Asia, noted that technology-based interventions had a beneficial effect on dietary behavior outcomes. The application of digital technology can aid in the designing and implementation of cost-effective interventions regarding the dietary behavior of adolescents.

### 4.1. Limitations

The change in intention status depends upon several external and environmental factors other than the constructs and the items in the TPB. Because of the focused use of the model framework, these exogenous variables were not accounted for in the study. As the participants' self-reported information on the constructs, there is a possibility of social desirability bias, despite the expressed confidentiality protocol.

## 5. Conclusion

Schools are necessary settings where children should develop behavior and skills for physical, emotional, and social well-being. The behavioral patterns developed during childhood and adolescence are continued, retained, and sustained into adulthood. The best predictor of behavior is behavioral intention which was used in the interventions in this study and demonstrated a statistically significant improvement in “Subjective norm” regarding healthy diet among the intervention group in the post-intervention phase. An expected finding was the improvement in intention following the intervention. Fostering healthy dietary behavior among school students through extra-curricular activities should be promoted. Any junk food stall around school premises should be discouraged by taking necessary steps. Appropriate advocacy with parents and school teachers is also essential; they should be sensitized and trained to understand and identify unhealthy dietary habits and preventive interventions.

## Data availability statement

The original contributions presented in the study are included in the article/Supplementary material, further inquiries can be directed to the corresponding author.

## Ethics statement

The studies involving human participants were reviewed and approved by Institutional Ethics Committee, All India Institute of Hygiene and Public Health. Written informed consent to participate in this study was provided by the participants' legal guardian/next of kin.

## Author contributions

SSJ and MD developed the concept and designed the data collection technique. AL and SSJ contributed to data collection, data validation, formal analysis, and preparation of the first draft. MD and CT provided the necessary supervision. All authors contributed to editing and developing the final version of the article.
