# Supplementary material for: School-based intervention on behavioral intention of adolescents regarding healthy diet in India
Source: Front Public Health. 2023 Feb 9;11:1094960. doi: 10.3389/fpubh.2023.1094960 (PMC9947774; doi:10.3389/fpubh.2023.1094960)
Supplement: Supplementary file 1 [file Data_Sheet_1.PDF]

Supplementary Material:

Study Questionnaire

Instruction to the respondents: The following are the questions that are pertinent to the research objectives. The respondents are requested to fill up the information as detailed below to the truest of their knowledge and belief. Instructions to fill up each section has also been provided. The respondents are requested to go through the instructions before filling-up each of the relevant sections.

Date:

Class: **ID code:**

Date of Birth/Age in years:

Gender: Male/ Female **Religion:** Hinduism/Islam/Others; if others then specify:

Type of family: Nuclear/Joint

BEHAVIORAL BELIEFS AND EVALUATION OF BEHAVIORAL OUTCOMES

Behavioral Beliefs

Instructions: The following statements are related to beliefs regarding dietary habits. Each of the following statements are expressions related to different issues on dietary habits of an individual. **Put a tick against "Agree" or "Disagree" columns for each statement** that you feel reflects your belief the most.

Item Code	Statement	Agree	Disagree
DB-1	Unhealthy diet will lead to lack of vitamins and minerals		
DB-2	Oily foods have harmful effects on health		
DB-3	Healthy diet does not provide sufficient protein		
DB-4	Healthy diet causes obesity/overweight		
DB-5	Healthy diet increases healthy life span		
DB-6	Healthy diet is tasteless		
DB-7	Healthy diet is expensive		

Evaluation of Behavioral Outcomes

Instructions: The following statements are related to the will incurred from the behavioral beliefs. The following statements reflect a scenario each. **Put a tick against "Good" or "Bad" columns for each statement** that you feel **reflects your situation** the most.

Item Code	Statement	Good	Bad
DBO-1	For me insufficient intake of vitamins and minerals on health is:		
DBO-2	For me effects of oily food on health is:		
DBO-3	For me insufficient protein intake is:		
DBO-4	For me obesity/overweight is:		
DBO-5	For me increase in healthy life span is:		
DBO-6	For me choice of food only on the basis of taste is:		
DBO-7	For me choice of food only on the basis of its cost is:		

NORMATIVE BELIEFS AND MOTIVATION TO COMPLY

Normative Beliefs

Instructions: The following statements are regarding important people around you, who may think that you should or should not eat healthy diet. **Put a tick against appropriate column of "Agree" or "Disagree"** that you feel reflects your belief the most.

Item Code	Statement	Agree	Disagree
DN-1	I think my mother wants me to regularly eat healthy diet		
DN-2	I think my father wants me to follow a healthy diet		
DN-3	I think my relatives/other family members want me to eat a healthy diet		
DN-4	I think my friends/peers do not want me to have healthy diet		
DN-5	I think my teachers want me to have healthy diet		
DN-6	I think the contents of television discourages me to have a healthy diet		
DN-7	I think the contents/discussions in the social media encourages me to regularly eat healthy diet		

<u>Motivation to Comply</u>

Instructions: The following statements are regarding motivation to perform certain activities related to dietary habits. **Put a tick against "Likely" or "Unlikely"** that you feel **reflects your appropriate motivational status**.

Item Code	Statement	Likely	Unlikely
**DMTC-1**	**I am motivated to eat healthy diet because my mother thinks I should eat healthy diet**		
**DMTC-2**	**I am motivated to eat healthy diet because my father thinks I should eat healthy diet**		
**DMTC-3**	**I am motivated to eat healthy diet because my relatives/other family members think I should eat healthy diet**		
**DMTC-4**	**I am not motivated to eat healthy diet because my friends/peers do not think I should eat healthy diet**		
**DMTC-5**	**I am motivated to eat healthy diet because my teachers think I should eat healthy diet**		
**DMTC-6**	**I am not motivated to eat healthy diet because television discourages me to eat healthy diet**		
**DMTC-7**	**I am motivated to eat healthy diet because contents/ discussions in the social media encourages me to eat healthy diet**		

CONTROL BELIEFS AND PERCEIVED POWER

<u>Control Beliefs</u>

Instructions: The following statements are related to beliefs on how & when control of dietary habits are exerted. **Put a tick against "agree" or "disagree" columns for each statement** that you feel reflects your belief in this case the most.

Item Code	Statement	Agree	Disagree
**DC-1**	**When hungry, it is very difficult for me to choose a healthy diet**		
**DC-2**	**If the taste of the food is good, I usually think of having them even if they are not healthy**		
**DC-3**	**When I am not depressed or sad, it is easy for me to eat a healthy diet**		
**DC-4**	**Even when junk food is available, I find it easy to opt for healthy diet**		
**DC-5**	**During celebrations and parties, it is easy for me to choose healthy diet**		
**DC-6**	**While visiting any mall, it is easy for me to choose healthy diet**		
**DC-7**	**While travelling, it is difficult for me to eat healthy diet**		

Perceived Power

Instructions: The following statements are related to the individual perception on how well the behavioral control beliefs are exerted. **Put a tick against "agree" or "disagree" columns for each statement** that you feel **reflects your situation** in this case the most.

Item Code	Statement	Agree	Disagree
**DPC-1**	**I can choose to eat healthy diet even during hunger**		
**DPC-2**	**I cannot choose to eat tasty food, if they are unhealthy**		
**DPC-3**	**I can choose to eat healthy diet even when I am depressed or sad**		
**DPC-4**	**I can choose to eat healthy diet even when junk food is easily available**		
**DPC-5**	**I can choose to eat healthy diet even during celebrations or parties**		
**DPC-6**	**I can choose to eat healthy diet even while visiting any mall**		
**DPC-7**	**I can choose to eat healthy diet even while travelling**		
